# On the Behavioural Biology of the Mainland Serow: A Comparative Study

**DOI:** 10.3390/ani10091669

**Published:** 2020-09-16

**Authors:** Sandro Lovari, Emiliano Mori, Eva Luna Procaccio

**Affiliations:** 1 Unità di Ricerca in Ecologia Comportamentale, Etologia e Gestione della Fauna–Dipartimento di Scienze della Vita–Università di Siena, Via P.A. Mattioli 4, 53100 Siena, Italy; moriemiliano@tiscali.it (E.M.); p.evaluna@gmail.com (E.L.P.); 2 Maremma Natural History Museum, Strada Corsini, 5, 58100 Grosseto, Italy

**Keywords:** aggressive behaviour, *Capricornis sumatraensis*, courtship, Rupicaprini, sociality

## Abstract

**Simple Summary:**

Serows *Capricornis* spp. are solitary, elusive, forest-dwelling goat-antelopes, allegedly the closest ancestral forms to wild sheep and goats (Caprinae). Their behaviour and ecology have been largely overlooked so far, although they could be useful to understand the roots of early ritualisation of weapons, i.e., horns. The activity rhythms, marking behaviour, and social interactions of captive mainland serows have been described and quantified. Activity peaked in mid-afternoon and late night, whereas resting and ruminating were the highest at noon and twilight. The two sexes used different marking sites and marking frequencies. A total of 33 social behaviour patterns were observed: 18 patterns concerned agonistic behaviour, whereas 15 patterns were relevant to courtship behaviour. An evolutionary comparison across Caprinae species with unritualised piercing horns, inclusive of serows, suggests that inter-sexual direct forms of aggressive behaviour are used significantly more often than indirect ones, except for chamois. Thus, Chamois *Rupicapra* spp. would be confirmed as the most advanced genus in terms of an early ritualisation of weapons, i.e., strongly hooked horns. Conversely, horns of the goral *Nemorhaedus* spp. and the serow lie on the same plane of the frontal bones, thus making possible the usage of a dominance display through frontal pushing.

**Abstract:**

Comparative behavioural studies help reconstruct the phylogeny of closely related species. In that respect, the serows *Capricornis* spp. occupy an important position as they have been assumed to be the closest forms to the ancestors of Caprinae. In spite of that, information on the behavioural repertoire of the mainland serow *Capricornis sumatraensis* is exceedingly poor. In this paper, we report data on the activity rhythms and social behaviour of rutting mainland serows in captivity (Central Thailand, January 1986; January–February 1987). Activity was bimodal with peaks in mid-afternoon and late night. Resting and ruminating peaked at noon and twilight. Four patterns of marking behaviour were observed out of a total of 1900 events. Males and females were found to use different marking sites and frequencies. A total of 33 social behaviour patterns were observed: 18 patterns concerned agonistic behaviour, whereas 15 patterns were relevant to courtship behaviour. A comparison across Caprinae species with unritualised piercing weapons (i.e., *Capricornis*, *Naemorhedus*, *Rupicapra*, *Budorcas*, and *Hemitragus*) has shown that inter-sexual direct forms of aggressive behaviour are used significantly more often than indirect ones, but for chamois, confirming *Rupicapra* spp. as the most advanced genus among them in terms of an early ritualisation of weapons. Conversely, horns of the goral *Nemorhaedus* spp. and the serow lie on the same plane of the frontal bones, thus making possible the usage of a dominance display through frontal pushing.

## 1. Introduction

Territoriality is a particularly important form of social behaviour, where an individual defends a particular area with limited resources, e.g., food, shelter, and mating partners, from conspecifics [1] (for a review). Noble (1939) defined a territory as “*any defended area*” [2], but later authors have emphasised the concept of intolerance and control within a geographically fixed spatial area [3] (for ungulates). Individuals perceive the presence of conspecifics through visual, tactile, auditory, or olfactory clues [4]. Differently from humans, olfaction plays an important role in the social behaviour of most mammalian species [5,6], including the perception of body odour of conspecifics, e.g., through mutual sniffing and scent-marking [7]. Territoriality usually involves the use of visual and olfactory signs, i.e., marking behaviour, on landmarks [8,9], and it is a “sit-and-wait” strategy, as opposed to “following” whereby a male follows foraging females [10]. Territory defence and access to potential sexual partners are the main reasons behind sociality [1,3].

Schaller (1977) and Walther (1984) defined as “direct forms of aggressiveness” (i.e., threats) those where weapons are oriented to target individuals or involving actual physical contact [9,11]. Conversely, indirect forms (i.e., displays) include the attempts of an individual to assert dominance by intimidating the opponent through the exhibition of physical vigour, body mass, and weapons. Competition among males of Caprinae has evolved through the ritualisation of aggressive patterns into mostly harmless ones, from direct to indirect forms [9,11,12]. No evidence of territoriality has been found within the Caprinae subfamily, with the exception of male chamois *Rupicapra* spp. [13,14]. It has been also suggested for male Arabian tahr *Arabitragus jayakari* [15], shown for both sexes of the Japanese serow *Capricornis crispus* [16,17], and there is conflicting evidence for gorals *Naemorhedus* spp. [18,19]. In the Japanese serow, mating takes place between individuals with overlapping territories and involves both submissive and aggressive patterns [17].

The mainland serow *Capricornis sumatraensis* Bechstein, 1799, is distributed from Sumatra to Indochina, Southern China, and Himalaya [20,21]. It is a solitary, forest ungulate, living at low densities and widely poached for meat and traditional medicine [22,23], thus being “vulnerable of extinction” *sensu* International Union for Conservation of Nature, IUCN [21,24]. In both sexes, two very large preorbital glands and a conspicuous olfactory apparatus suggest an intensive use of scenting in communication [22,25]. Detailed data on its ecology are very poor [26,27,28,29], and information on its behavioural repertoire is absent, although the mainland serow is assumed to be close to the ancestor of Rupicaprini species [12,20], and information on its social behaviour can be useful to understand the evolution of behaviour within the Rupicaprini tribe. Lovari et al. (2009) stated that “the shape/size of weapons and the type of fighting are related. Caprini males have developed large, ritualised horns adapted to clash and butt which may indicate the fighting potential of their bearer, whereas Rupicaprini have non-ritualised, stiletto-like horns suited to gore the opponent and when used on an opponent, they may induce a retaliatory attack and sometimes even break during the fight” [30].

We have attempted to describe qualitatively and quantitatively the activity rhythms, marking behaviour and social interactions (i.e., courtship) of the mainland serow, under captive conditions. A comparison of the behavioural repertoires within the Tribe Rupicaprini, as well as other Caprinae with non-ritualised weapons in both sexes (takin *Budorcas taxicolor*; Himalayan tahr *Hemitragus jemlahicus*), has also been conducted.

## 2. Materials and Methods

### 2.1. Study Area

**Figure 1:**
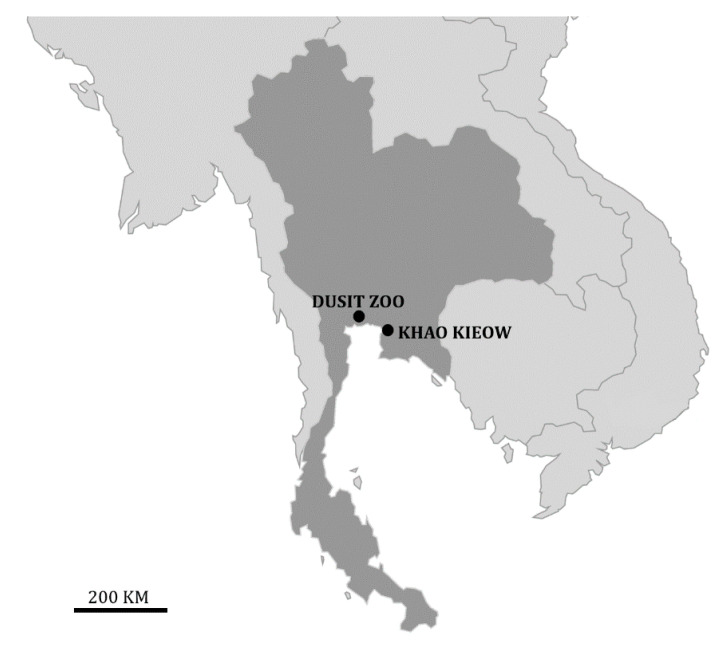
Location of the study sites (Thailand).

Our study was conducted on two pairs of adult (≥4 years old) serows (1 adult male, 1 adult female, for each pair), each of them in a fenced wildlife site, in Central Thailand (Figure 1). Members of each pair were born in the wild, but they were kept together in captivity in the preceding 2−3 years. We are aware of our limited sample size (*n* = 2, for each sex), which makes our results in need of confirmation on a larger sample of serows. Captive mainland serows are available in just a few zoological gardens in Asia but not elsewhere (e.g., https://zooinstitutes.com/animals.html; http://www.zootierliste.de/en). Anecdotal information reports that both sexes are intolerant of other individuals of the same sex within the same enclosure [31], which may explain why serows are not found in social groups larger than pairs in captivity.

The Khao Kheow Wildlife Sanctuary (11°12′ N–101°30′ E) was about 20 km southeast of Chonburi. The other site (13°46′ N–100°30′ E) was located in a zoo precinct (Dusit Zoo, in Bangkok). Both enclosures (about 10,000 m^2^ in Khao Kheow; ca. 200 m^2^ in Dusit Zoo) included open areas, trees, shrubs, and an artificial shelter. Water was available full time, whereas some food was provided once a day, at about 09:30 a.m.–10:00 a.m. in both areas. Additional food was provided haphazardly during the day by park visitors.

The climate of Thailand is tropical and strongly influenced by the monsoons. Annual rainfall is about 1400 mm, peaking in May–September [32]. The average maximum annual temperature is 36 °C in April; the average minimum is 16 °C in January. In Thailand, free-living mainland serows inhabit steep limestone mountains and cliffs thickly covered with forest that is inaccessible to man and with rocky hollows used by serows as shelter sites [22].

### 2.2. Data Collection

Serows were watched during the day (06:30 a.m.–01:30 p.m.; 02:00 p.m.–06:30 p.m.) and, twice per week round-the-clock (including from 06:30 p.m. to 06:30 a.m.), at a distance of ca. 15–40 m, through binoculars (©Leitz, Wetzlar, Germany, 10 × 40 field glasses, during the day; ©Zeiss, Jena, Germany, 8 × 56 BT*P* during the night) whenever details should be recorded. Observations were conducted by two operators at the same time, with different tasks (see below), for a total of 166 h in January 1986 (Dusit Zoo), and for 98 h in January–February 1987 (Khao Kheow Wildlife Sanctuary), during the rutting season. One observer concentrated on all marking activities, reporting onto check-sheets the day, time, mark type, the exact location within the enclosure where marking occurred, and the sex of the marking individual; the other observer recorded all behavioural observations, except for marking. Behaviour patterns and activity rhythms were recorded onto paper check-sheets.

### 2.3. Data Analysis

Patterns of activity rhythms and relevant 95% confidence intervals were estimated through the software R 3.6.1 (version 3.5.1., R Foundation for Statistical Computing, Wien, Austria), package “overlap” [33].

The occurrence of each behavioural type was considered as a percentage of occurrence (frequency). The intersexual difference in the frequency of marking activities, which may support territorial behaviour [6], was tested through a Fisher’s exact test. The one-sample Kolmogorov–Smirnov (K-S) test was used to assess if each serow marked sites with the same frequency. Two-sample K-S tests were used to evaluate whether males and females of each pair made use of the same marking sites. Both tests were performed through the software IBM SPSS Statistics 21 [34].

For each study site, the behavioural repertoires of males and females were compared within and between sexes through the Fisher’s exact test. If the intrasexual difference between two behavioural repertoires was not statistically significant, we pooled data together to obtain one behavioural repertoire per sex. We used a Z-test to assess differences between the proportions of direct and indirect forms of aggression by serow and other closely related species observed in the wild: the Himalayan goral *Naemorhedus goral* [19], the Northern chamois *Rupicapra rupicapra* (Lovari and Albicocco, unpublished data), the Southern chamois *Rupicapra pyrenaica* [35], the Himalayan tahr [36], and the takin (Lovari and Dahal, unpublished data). All studies included in our comparison were performed by one of us (SL) to reduce observer’s bias. For each species, intrasexual differences were compared through Z-tests, whereas differences between sexes belonging to the same species were tested through the Fisher’s exact test. The Z-test, the K-S test, and Fisher’s exact test are suitable to small sample sizes [37,38].

## 3. Results

### 3.1. Activity Rhythms and Marking Behaviour

**Figure 2:**
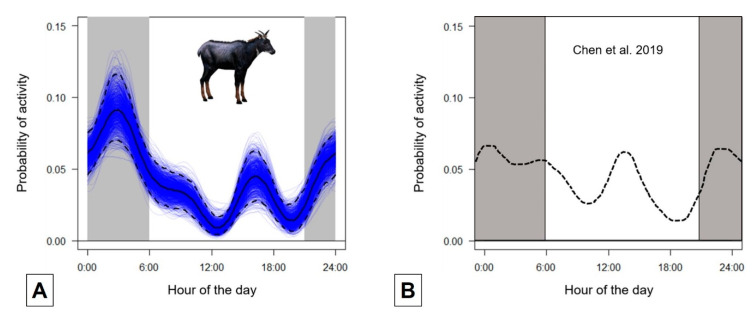
(**A**) Activity patterns of captive mainland serow, expressed as kernel density estimates throughout the 24 h (*n* = 152 records). The solid black line shows the average trend, dashed black lines represent 95% confidence intervals. Blue lines show bootstrapped estimates of activity patterns. (**B**) Activity patterns of free-ranging mainland serow (from [23], modified).

The activity pattern was bimodal with two peaks, one in the mid-afternoon and the other in the second half of the night, whereas resting and ruminating peaked at noon and twilight (Figure 2A).

**Figure 3:**
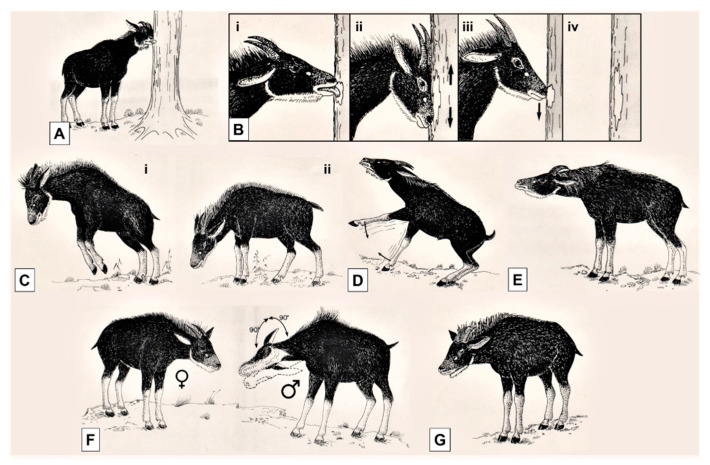
Behavioural repertoire of the mainland serow. Marking with (**A**) preorbital gland, with licking; (**B**) forehead (**i**, licking; **ii**, forehead rubbing; **iii**, scent sniffing; **iv**, eventual mark); (**C**) Head Down (**i**) first phase, with fore-feet stamping (**ii**) second phase, freezing in Head Down; (**D**) Foot Stamping; (**E**) Low Stretching; (**F**) Twisting (male), with female standing in Staring to the male; (**G**) Staring.

Four types of marking behaviour were observed for a total of 1900 events (preorbital gland, 70% and 90%; forehead/nape, 15% and 5%; horns, 10% and 4%; flank, 5% and 1%, respectively in Dusit Zoo and Khao Kheow) performed by both the male and the female of each pair. In a typical sequence of scent marking, serows sniffed a spot, lay scent there, and alternated marking with licking movements (Figure 3A,B).

As to the pair in Dusit Zoo (hereafter, pair 1, composed by male M1 and female F1), the male was responsible for 80.5% of total marking events (N_1_ = 1714) (Fisher’s Exact Test = 638.34, dof = 1, *p* < 0.0001). In Khao Kheow (hereafter, pair 2, composed by male M2 and female F2), the male accounted for 58.0% of the events (N_2_ = 186) (Fisher’s Exact Test = 1.742, dof = 1, *p* = 0.187).

The marking activity was mainly carried out through preorbital glands by both sexes (Table 1). The intensity of marking activity sharply declined in both sexes in the post-oestrus days of the females (Figure S1).

In pair 1, the male and the female marked 42 and 22 sites respectively, whereas, in pair 2, the male and the female marked respectively 17 and 27 sites. In each pair, males and females were found to use different sites and marking frequencies (K-S tests: 2.26–14.71, *p* < 0.0001).

### 3.2. Behavioural Repertoire

A total of 33 social behaviour patterns were observed: 18 patterns concerned agonistic behaviour (Table 2), whereas 15 patterns concerned courtship behaviour (Table 3).

Direct and indirect forms of aggression, as well as socially explorative behaviour built up 24% and 9% of the behavioural repertoire of M1 and M2 respectively, whereas courtship patterns were predominant (M1, 76%; M2, 91%).

Behavioural repertoires of females differed slightly from each other. Direct forms were the most common in the behavioural repertoire of F1 (71%), followed by indirect forms (24%) and socially explorative behaviours (5%). F2 performed mainly direct aggressive forms (43%), followed by courtship patterns (27%), socially explorative behaviours (25%), and indirect forms (5%).

The most frequent behaviours were Kicking, Head Up and Mount for males, and Head Down, Licking Genitals/Urine, and Stare for females (Table 4). As to the intersexual behaviour, the most frequent behaviour was Head Down for both males (53%) and females (46%), followed by Approaching for males (23%) and Staring for females (20%). During courtship, Kicking was performed most frequently by males (M1, 47%; M2, 54%), followed by Head Up for M1 (16%) and Licking Genitals/Urine for M2 (17%).

### 3.3. Direct and Indirect Forms

**Figure 4:**
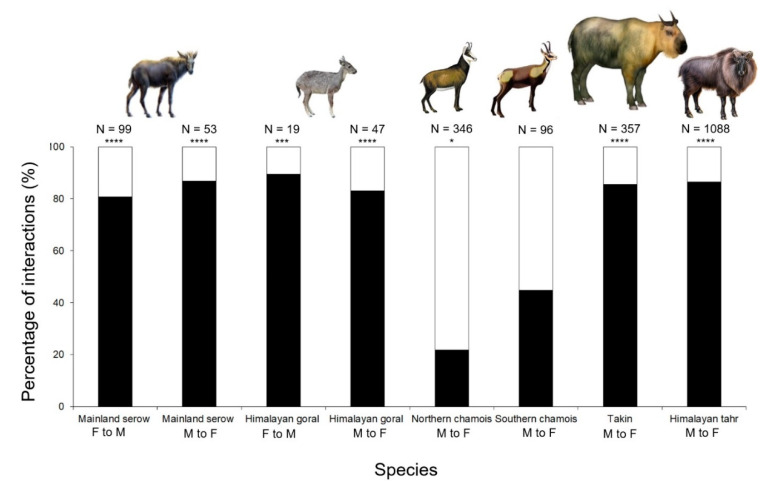
Direct and indirect forms of intersexual aggressive behaviour. Significance levels (Z test): * *p* < 0.05; *** *p* < 0.001; **** *p* < 0.0001. Black = direct forms; white = indirect forms. For sources, see Material and Methods—Data Analysis. M = male; F = female. N = number of events.

Males and females of Caprinae species with piercing weapons were considered in Figure 4. Only chamois performed direct forms of aggressive behaviour significantly more than indirect forms (Figure 4; Table 5). There was no significant difference between sexes (Fisher’s exact test, mainland serow: *p* = 0.498; Himalayan goral: *p* = 0.71) (Figure 4).

## 4. Discussion

Our data show two bimodal peaks of activity, a nocturnal one at ca. 03:00 a.m. and a diurnal peak at ca. 04:00 p.m, in contrast to what has been believed so far of the serow as a mostly nocturnal mammal [21,22,40,41]. In fact, diurnal activity of mainland serows was also recorded by [23] in the wild (Figure 2B). The activity patterns we recorded in captive conditions are consistent with those recorded by Chen et al. [23], which suggests that confinement in captivity and artificial availability of food resources have not altered their basic activity rhythms. One could object that mainland serows seem to show a few adaptations to nocturnal life, e.g., blackish to black coat colour, acute senses of smell and hearing [22]. However, all these features could also have evolved as adaptations to life in dark habitats, such as dense forests [29].

Large nostrils and particularly big preorbital scent glands, as well as the usage of latrines as territorial landmarks, suggest that this species relies heavily on olfactory signals [22,25,31,42], which would be useful for communication in conditions where visual ones may not be perceived.

Enclosure size may have elicited a different frequency of marking and aggressive activities [43,44], but we detected no other qualitative difference between study sites. In spite of our limited sample size and of captive conditions, a few preliminary conclusions may be drawn. Although intense marking was more evident in the male sex, females showed it too, confirming the prediction of territorial marking in both sexes [8,9]. Most marking occurred using the secretion of preorbital glands, as it has been reported for the Formosan serow *C. swinhoei* [42]. Serows are sexually monomorphic, also as to horn size and shape: thus, males should be cautious when approaching females, especially in the reproductive period. In fact, females who are not yet fully receptive may be quite aggressive to males, to the point of wounding them seriously (see [45], for the closely related mountain goat *Oreamnos americanus*). Amongst animals, direct attacks are usually avoided to limit the risk of being physically injured [46,47,48]. The dagger-like, sharply-pointed and potentially lethal horns of serows may have promoted the development of alternative tactics, e.g., marking behaviour and dominance displays, with respect to actual fights. The spatial pattern and the usage of different marking sites for each sex suggest the existence of individual preferred locations, as well as the absence of shared marking sites [17,42].

Intersexual tolerance increased with the oestrus development of the females when the intensity of their marking activity sharply declined (Figure S1). Kicking was the most used courtship behaviour pattern by males, as it is in the other serow and goral species [17,19,42]. Females may react to Kicking with urination, which provides males with olfactory cues of the female oestrus status [11,49]. The simulation of infant behaviour (i.e., Low-Stretching) is the most used courtship pattern by male serows approaching oestrus females, as in other Caprinae species [9,11,33,50]. The presence of a single male serow in each enclosure determined no competition to female access, thus ruling out the opportunity to record intra-sexual male agonistic behaviour.

The Rupicaprini tribe includes serows, gorals, the mountain goat, and chamois, which are all nearly monomorphic species, but for temporary (chamois) or permanent (mountain goat) body mass dimorphism. The takin (Tribe Budorcatini) and the Himalayan tahr (Tribe Caprini) show some Rupicaprini traits, i.e., they are little sexually dimorphic as to the shape and size of horns.

Among Rupicaprini, the Twist is present—although rare—only in the mainland serow, whereas it is often associated to Low Stretching and Kicking in the bharal *Pseudois nayaur* [51], as well as in *Capra* and *Ovis* spp. [11]. In this Tribe, Frontal Pushing has been reported only for serows, although it has been recorded often in other species of the Caprini Tribe, e.g., the Himalayan tahr [36], the takin [52], and the Mediterranean mouflon *Ovis aries* [53]. Therefore, both the Twist and Frontal Pushing—rare or absent in Rupicaprini—become much more abundant in the phylogenetically most advanced Caprinae (Figure S2). Aggressive behaviour builds up over 80% of the repertoire of the mainland serow, and direct forms of aggression are significantly more used than indirect ones. Serows are relatively close to the ancestors of Caprinae species [20]. Aggressive behaviour may be expected to be less differentiated in these phylogenetically ancestral forms of resource defenders, which have not yet developed ritualised weapons, e.g., those of *Pseudois, Capra,* and *Ovis* spp. [12]. Beside mainland serows, also gorals show mostly direct aggressive displays [19], whereas chamois, i.e., the most recent genus among Rupicaprini [20,54], may have developed a repertoire privileging indirect forms of aggressiveness. Among Rupicaprini, intrasexual aggressiveness by females is comparable to that shown by males, with some differences in phenology (Southern chamois [55] and mountain goat [56]). Body size and horn size are not different between male and female gorals and serows. The frequent use of intersexual direct forms has also been found in male Himalayan tahrs and takins [30,36], which have not evolved ritualised weapons as *Ovis* and *Capra* spp.

Lovari (1985) suggested that there could be a direct relationship between well-developed social behaviour and the complexity of behavioural repertoire [35]. This suggestion has not been confirmed through our study. The genus *Capricornis* was the first to diverge within the Rupicaprini clade, thus qualifying as the most ancestral taxon [20,54]. We recorded 29 behaviour patterns for the mainland serow, as many as those observed in both chamois species, much less solitary ungulates than serows, but more than in the Japanese serow and in the Himalayan goral [17,19].

Among Rupicaprini, Frontal Pushing has been recorded only in serows, whereas it is used rarely by the Himalayan tahr and frequently by the takin and other Caprinae [11,36,47,52]. Conversely, the Head Down is common to all Caprinae species with small horns, including serows, chamois, Himalayan tahr, and takin [11,50]. In all these species, the Head Down is a threat where horns are usually oriented to the target individual: conversely, among sheep *Ovis* spp. and goats *Capra* spp., it is a rare behaviour and mostly an indirect display [11]. The mainland serow performs Head Down statically by hitting the fore hooves on the ground in unison. This behaviour increases in dynamism in the Himalayan goral and in the chamois, which perform it while moving and keeping down their head [19,55].

## 5. Conclusions

Among Rupicaprini, the most efficient weapons are those of the mountain goat, which are stiletto-like with an excellent inclination to stab an opponent [50]. Conversely, the horns of the goral and the serow lie on the same plane of the frontal bones, thus making possible the usage of an alternative dominance display (i.e., Frontal Pushing). The high proportion of indirect forms of aggressive behaviour in both species of chamois suggests *Rupicapra* spp. as the most advanced genus among those considered in Figure 4 [20,54]. In fact, horns with hooked tips are unlikely to be efficient weapons, as the hook prevents a direct stab and forces to strike at an odd angle. Furthermore, thinly shaped hooks break easily inside the body muscles of an opponent [56].

Chamois are the most social Rupicaprini, and increasing gregariousness is likely to generate a change in weapon system from “daggers” to—ultimately—wrestling-type weapons [56]. Therefore, chamois may have evolved hooked horns, without yet having developed wrestling- or butting-type horns and behaviour, cf. *Capra* spp.

Lovari and Apollonio (1994) argued that sociality may have triggered the evolution of ritualised horns within the Rupicaprini Tribe [19]. Yet, if so, no evolutionary push to develop a ritualised usage of horns should have occurred in the solitary serow. While detailed information on the social organisation of the mountain goat and of both chamois species is available [57,58,59], we still lack detailed information on the social behaviour of the other Caprinae with near-monomorphic horns between the two sexes. This information is crucial to further develop hypotheses on the evolution of aggressive behaviour, in turn horn shape and size, of little dimorphic caprids.

## Figures and Tables

**Table 1:** Occurrence of different mark types. M1–F1: pair of Dusit Zoo, M2–F2: pair of Khao Kheow. *n* = number of observations, % = percentage of the total.

Marking Type	M1		F1		M2		F2	
	*n*	%	*n*	%	*n*	%	*n*	%
Preorbital Gland	1216	88.1	258	77.3	90	88.2	38	45.2
Forehead	125	9.1	34	10.2	5	4.9	12	14.3
Horn/Nape	13	0.9	17	5.1	6	5.9	19	22.6
Flank	26	1.9	25	7.5	1	1	15	17.9
Total	1380		334		102		84	

**Table 2:** Behavioural repertoire of the mainland serow: I, indirect form of aggression; D, direct form of aggression. M, male; F, female. See also Table 4.

Behaviour Pattern	Description
Approaching (M, F)	Threat. The sender walks straight to the receiver. The mane may be erected (D).
Body/Head Shaking (F)	Dominance display. The sender vigorously shakes the head several times (I).
Chasing (F)	Threat. The female chases the male for a few metres. Performed only by F1 (D).
Clashing (M, F)	Threat. Two individuals front each other, with lowered head, and clash after a short rush. Sometimes alternated with Front Pushing (D).
Conflict Posture (M)	Dominance display. It shows elements of submissive behaviour mixed to dominant ones; mouth may be slightly open; ears may be turned outwardly, slightly drooping (I).
Frontal Pushing (M, F)	Threat. Two individuals move their foreheads into contact and start pushing (D). Sometimes preceded by Head Down (D).
Gamboling (M, F)	Dominance display. A downhill run that includes vigorous head nodding, alternately lifting up forequarters and hindquarters (I).
Head Butting (M, F)	Threat. An individual butts the opponent’s body with its forehead (D).
Head Down (M, F)	Threat (Figure 3C). An individual holds its head lower than main axis of body, pointing horns to another one. The mane is erected. It may be performed laterally or frontally (D).
Hooking (M, F)	Threat. One attempts to gore another with a horn sweep. It may be frontal (to shoulder/neck of opponent) or side-on (to rump/abdomen of opponent) (D).
Horning Vegetation (M, F)	Dominance display. An individual horns a bush vigorously and/or lay scent on it (I).
Licking Body (M, F)	Socially explorative behaviour [9]. An individual licks the other’s body. Licked areas are generally muzzle, flank, and genitals.
Naso-Nasal Contact (M, F)	Socially explorative behaviour. An individual touches the nasal region of the other with its nose.
Rushing (F)	Threat. An individual rushes quickly to the other one. Head position may vary. Performed only by F1 (D).
Side Display (M)	Dominance display (I). An individual stands on stiffly stretched legs, broadside on; back is hunched, head is lowered as in Head Down (D).
Sniffing Forehead/Hoof (M, F)	Socially explorative behaviour. An individual sniffs glands of the other. Sniffing may help partner recognition.
Snorting (F)	Threat. A sound uttered by a violent puff through nostrils, mainly during Chasing and Rushing (D).
Staring (M, F)	Threat (Figure 3G). An individual stares the other one, with mane erected (D).

**Table 3:** Behavioural repertoire of the mainland serow; male and female behaviour patterns in courtship.

Behaviour Pattern	Description
Courtship Foot Stamping (M)	Male stamps the ground with one of his fore-hooves, sometimes quickly alternating with both of them, behind the female. Mane can be erected (Figure 3D).
Croup Touch (M)	Male rests his chin, throat, and lower part of the neck on the female’s croup. Often preceded or followed by Kicking.
Flank Stroke (M)	The male softly strokes the female’s hindquarters with the inner surface of one of his forelegs in a downward sweep.
Following (M)	Male follows female closely.
Head Up (M)	Male approaches the female with stiff legs, neck raised, and uplifted muzzle. Tongue flicks may be performed by the male.
Kicking (M, F)	An individual, the male generally kicks the other’s flank, abdomen, or hind-legs with a stiff foreleg. It may be performed frontally, laterally, or from behind.
Licking Genitals/Urine (M, F)	An individual licks the other one’s genitals or urine. Individuals can lick each other simultaneously. It may help male to verify female oestrus stage through her pheromone odour.
Lip-Curling (M)	Male sniffs female’s vulva or urine, slowly lifting his muzzle and curling the upper lip. It helps scent perception by activation of the Jacobson’s organ.
Low Stretching (M)	Submissive display. Male approaches female with quick steps—or it may stand still—on flexed carpal joints, with a lowered and outstretched neck, with chin slightly raised. Horns partly concealed in mane (Figure 3E).
Mounting (M)	Copulative behaviour. Female stands with rear legs slightly spread and tail upraised, while male rises up on hindlegs and partly rests on top of female. Not all mounts end up in copulations, as female may withdraw (cf. Intentional Mounting).
Intentional Mounting (M)	Copulative behaviour. Male attempts to mount female, unsuccessfully.
Naso-Genital Contact (M)	Male sniffs the genital region of the female.
Sniffing Urine (M)	Male sniffs the area where female has urinated. Usually, Lip-Curling follows.
Tongue Flicking (M)	Male, behind female, from the Low Stretching posture, flicks out his tongue repeatedly, with head and neck in Low Stretching.
Twisting (M)	The male lowers his neck into a position that looks like the Low Stretching and then rotates his head up to 90° so that horns face away from the female (Figure 3F).

**Table 4:** Occurrence of behaviour patterns of the mainland serow. M1–F1, pair of Dusit; M2–F2, pair of Khao Kheow. Only the sex of the sender is indicated, as the animals were kept in heterosexual pairs.

Behaviour Pattern	Sender			
	% M1	% M2	% F1	% F2
Approaching	2.8	1.3	3.2	1.2
Body/Head Shaking	0.0	0.0	7.9	0.0
Chasing	0.0	0.0	1.6	0.0
Clashing	1.4	0.5	1.6	3.7
Conflict Posture	1.4	0.0	0.0	0.0
Croup Touching	0.0	5.0	0.0	0.0
Flank Stroking	0.0	1.6	0.0	1.2
Following	0.7	0.5	0.0	0.0
Foot Stamping	3.5	1.6	0.0	0.0
Frontal Pushing	2.8	0.5	3.2	0.0
Gamboling	0.0	0.3	11.1	3.7
Head Butting	0.0	0.3	0.0	4.9
Head Down	7.8	2.6	20.6	21.0
Head Up	12.1	2.1	0.0	0.0
Hooking	0.0	0.5	7.9	7.4
Horning Vegetation	0.7	0.0	4.8	1.2
Intentional Mounting	0.7	6.3	0.0	0.0
Kicking	36.9	28.2	0.0	1.2
Licking Body	0.7	0.0	0.0	1.2
Licking Genitals/Urine	3.5	7.4	0.0	25.9
Lip-Curling	5.0	0.8	0.0	0.0
Low Stretching	6.4	1.6	0.0	0.0
Mounting	0.0	29.7	0.0	0.0
Naso-Genital Contact	5.0	2.9	0.0	0.0
Naso-Nasal Contact	2.1	1.8	4.8	9.9
Rushing	0.0	0.0	11.1	0.0
Side Display	0.0	0.8	0.0	0.0
Sniffing Forehead/Hoof	2.8	0.3	0.0	13.6
Sniffing Urine	2.1	1.1	0.0	0.0
Snorting	0.0	0.0	4.8	1.2
Staring	1.4	0.0	17.5	2.5
Tongue Flicking	0.0	1.3	0.0	0.0
Twisting	0.0	1.1	0.0	0.0

**Table 5:** Comparison of the behaviour patterns recorded for adult mainland serow, Japanese serow [16,17,39], Himalayan goral, Northern chamois, Southern chamois, Himalayan tahr and takin. +, present; (+), rare (<1% of occurrence); ?, dubious; -, absent. Unless indicated otherwise, references are those listed in Material and Methods—Data Analysis.

Behaviour Pattern	Mainland Serow	Japanese Serow	Himalayan Goral	Northern Chamois	Southern Chamois	Takin	Himalayan Tahr
Approaching	+	-	+	+	+	+	+
Blocking	-	-	-	-	-	-	+
Body/Head Shaking	(+)	-	(+)	+	+	(+)	+
Bunting (Mock-Suck)	-	-	-	-	+	-	-
Chasing	(+)	+	+	+	+	+	+
Clashing	+	+	-	(+)	(+)	-	+
Conflict Posture	(+)	?	-	-	+	-	-
Croup Touching	+	+	+	?	-	+	+
Flank Stroking	(+)	-	+	-	+	(+)	-
Following	(+)	+	+	?	?	+	-
Foot Stamping/Herding	+	-	-	+	-	-	+
Frontal Pushing	+	+	-	-	-	+	+
Gamboling	+	-	+	+	+	-	-
Head Butting	(+)	+	+	+	+	-	+
Head Down (Static)	+	+	+	+	+	-	+
Head Down (Dynamic)	-	-	+	+	+	-	+
Head Up	+	+	+	+	+	-	-
Hopping	-	(+)	-	+	+	-	-
Hooking	+	+	+	+	+	-	+
Horning/marking Vegetation	(+)	+	+	+	+	+	+
Humping	-	-	-	-	-	-	+
Intentional Mounting	+	+	+	+	+	+	+
Kicking	+	+	+	(+)	-	+	-
Licking body	+	+	+	+	+	-	-
Licking genitals/urine	+	+	+	+	+	+	+
Lip-Curling	+	+	+	+	+	+	+
Low Stretching	+	+	+	+	+	-	+
Mounting	+	+	+	+	+	+	+
Naso-Genital Contact	(+)	+	+	(+)	(+)	+	+
Naso-Nasal Contact	+	+	+	(+)	+	+	+
Neck-Fighting	-	-	-	+	(+)	+	-
Neck-Up	-	-	+	+	+	+	-
Penile Display	-	-	-	+	+	+	+
Poking	-	-	-	+	-	-	+
Rushing	+	(+)	+	+	+	+	+
Side Display	(+)	-	-	+	+	+	-
Snorting	+	+	+	+	+	+	-
Sniffing Forehead/Hoof	+	+	-	-	-	-	-
Sniffing Urine	+	-	-	-	-	-	+
Staring	(+)	-	+	+	+	+	+
Male Urinating in Female Posture	-	-	-	+	+	-	-
Tongue Flicking	(+)	-	-	-	-	-	+
Twisting	(+)	-	-	-	-	-	-
TOTAL	33	23	26	31	31	22	27

## Supplementary Materials

The following are available online at https://www.mdpi.com/2076-2615/10/9/1669/s1, Figure S1: Frequency of marking events within the oestrus period. M1–F1: pair of Dusit Zoo, M2–F2: pair of Khao Kheow, Figure S2: Neighbour-Joining phylogenetic tree of total mtDNA consensus sequences of Rupicaprini and Caprini genera included in our comparison. Numbers at nodes indicate bootstrap values. Sequences were retrieved from GenBank (*Capricornis*: FJ207534, NC_020629, KU605670, KF856568, KT345703, MK303945, NC_010640, NC_012096 and AP003429; *Naemorhedus*: KP203894, FJ469673, FJ207532, KT878720; *Rupicapra*: NC_020633, KJ184173; *Hemitragus*: FJ207531; *Budorcas*: MH049869, MK748332). The giraffe *Giraffa camelopardalis* (sequence NC_024820) was used as outgroup.
