# Supplementary material for: On the Behavioural Biology of the Mainland Serow: A Comparative Study
Source: Animals (Basel). 2020 Sep 16;10(9):1669. doi: 10.3390/ani10091669 (PMC7552253; doi:10.3390/ani10091669)
Supplement: Supplementary file 1 [file animals-10-01669-s001.pdf]

Supplementary file

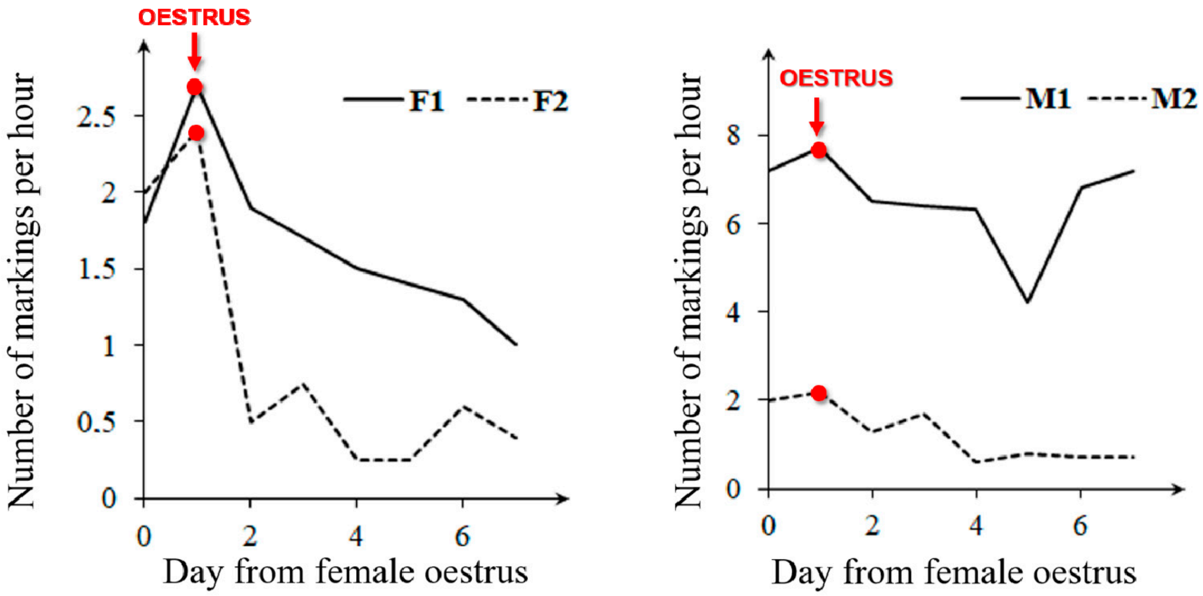

Figure S1. Frequency of marking events within the oestrus period. M1 – F1: pair of Dusit Zoo, M2 – F2: pair of KhaoKheow.

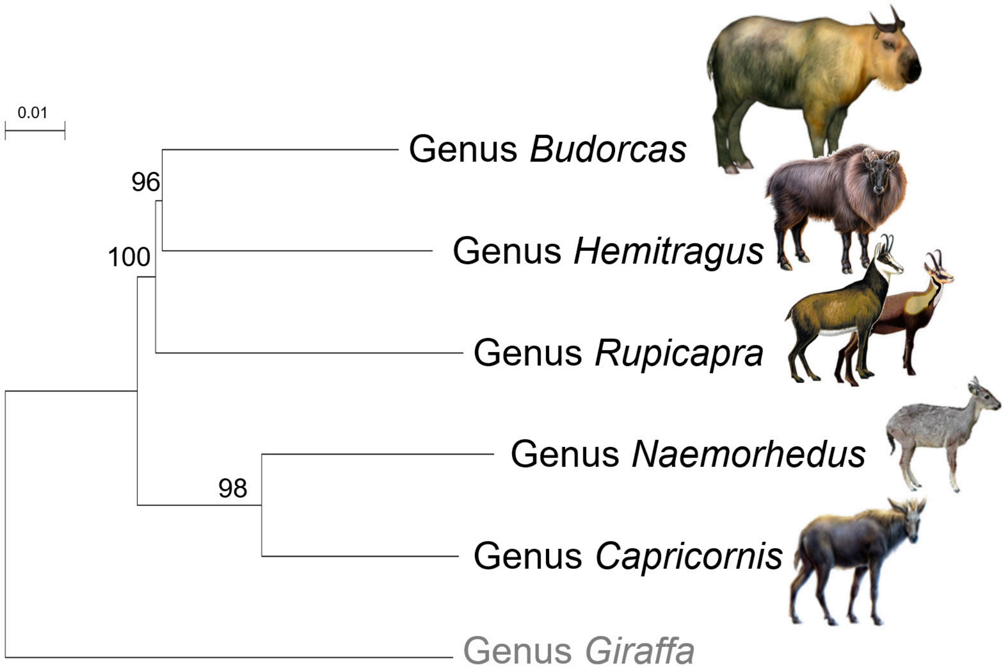

Figure S2. Neighbour-Joining phylogenetic tree of total mtDNA consensus sequences of Rupicaprini and Caprini genera included in our comparison. Numbers at nodes indicate bootstrap values. Sequences were retrieved from GenBank (*Capricornis*: FJ207534, NC_020629, KU605670, KF856568, KT345703, MK303945, NC_010640, NC_012096 and AP003429; *Naemorhedus*: KP203894, FJ469673, FJ207532, KT878720; *Rupicapra*: NC_020633, KJ184173; *Hemitragus*: FJ207531; *Budorcas*: MH049869, MK748332). The giraffe *Giraffacamelopardalis* (sequence NC_024820) was used as outgroup.
